# Antioxidants in Male and Animal Reproduction: Applications and Critical Issues

**DOI:** 10.3390/antiox13111283

**Published:** 2024-10-23

**Authors:** Elena Moretti, Cinzia Signorini

**Affiliations:** Department of Molecular and Developmental Medicine, University of Siena, 53100 Siena, Italy

## 1. Introduction

The Special Issue “Antioxidants in Male Human and Animal Reproduction: In Vitro and In Vivo Studies”, published by *Antioxidants* and led by us (https://www.mdpi.com/journal/antioxidants/special_issues/Male_Human_Animal_Reproduction, accessed on 21/10/2024), represented the opportunity to collect—through seven research papers and two review articles—recent scientific findings on the effects of oxidative stress (OS) and antioxidants on spermatogenesis, semen quality, and semen cryopreservation processes. Studies on humans and animal models have proven to be scientifically sound in providing updates and indications of innovative applications on the topic of the aforementioned Special Issue.

Infertility represents a relevant worldwide concern affecting about 40–50% of couples around the world, with the male factor accounting for about 50% of the cases [1].

One of the most important recent acquisitions in the field of male infertility is the relevant role of OS, which can be a causative factor in 30–80% of infertile men [2]. Indeed, OS has emerged as a common finding in many reproductive pathologies such as varicocele, genitourinary infections, and inflammation, as well as in cases of exposure to environmental toxicants and heavy metals and in the presence of uncorrected lifestyles such as smoking [3].

OS-induced male infertility represents an issue of great concern for clinicians and scientists because, from a clinical perspective, OS is a contributing agent to reduced fertilization rates and overall success rates of assisted reproductive technologies (ARTs).

In the male infertility field, as in all research areas, the evaluation of OS requires increasingly accurate measurements and clear indications of which aspect of OS is being evaluated, for example the reduction/damage/deficiency of antioxidant defenses, the effect of antioxidants, or oxidative damage. In the latter case, it is necessary to specify the type of molecules to which the oxidative damage evaluated is attributable, and it is important that such studied molecules are relevant to the pathophysiological processes of the analyzed conditions. Interestingly, the relevance of biomarkers of oxidative damage in male infertility can be useful to classify subjects, evaluate the progression of the condition, or evaluate the effects of integrating antioxidants. In particular, the effects of antioxidants are a relevant topic in cryopreservation. In recent decades, many studies have focused on methods and strategies able to improve the freezing–thawing efficiency of semen, a main issue in the field of human and animal assisted reproduction. In particular, animal semen freezing is even more widespread and contributes to genetic improvement through artificial insemination, supporting biodiversity conservation and the preservation of endangered species [4]. Cryopreservation can have many benefits but can impair sperm function. Engineering and supplementing media with antioxidants is a popular strategy to overcome this problem. Also, during semen manipulation, several factors such as centrifugation, deprivation of seminal plasma containing the main antioxidant compounds, and others can cause OS. Also in these cases, a mitigation strategy with antioxidants can reduce reactive oxygen species (ROS) production and consequently OS [5]. On this subject, the literature requires updated reviews and categorization of the doses and types of antioxidants used. In this regard, antioxidants intended as in vivo or in vitro supplements should be distinguished: antioxidant supplementation can be used on patients with established protocols or in the semen used in the laboratory procedures applied both in research field and during ART.

Last but not least, animal models of different species have been used to study the efficacy of antioxidant compounds in OS conditions, analyzing several compounds including nanoparticles or antioxidant-based diets [6,7,8,9]. In this regard, studies on living species, even far from human ones, can represent the opportunity to investigate specific molecular mechanisms shared between several living species and therefore enable the initiation of studies that have repercussions on human clinical practice. In addition, it is also worth noting that some animal models can represent the opportunity to investigate the effects of altered spermatogenesis and reproduction on ecosystems. Furthermore, the effects, on male fertility, of molecules modulating the antioxidant response and applied in the treatment of chronic diseases are increasingly worthy of attention.

The topics covered in the Special Issue are discussed below.

## 2. Human Clinical Studies

The review of Alfaro Gómez et al. [10] excellently highlights how the relation between OS and male infertility becomes more and more clear. New terms such as “Male Oxidative Stress Infertility” have emerged to indicate a new category of infertile men with high OS levels, who until now have been included in the idiopathic group. The review discusses the debated use of antioxidants in treating infertile patients and the possibility that the use of antioxidants can avoid the necessity of assisted fertilization techniques [11], even though evidence-based antioxidant treatments that directly improve seminal parameters or birth ratio are still lacking. The questionable efficacy of antioxidants at high doses remains an uncertainty that opens a window for new studies. In particular, for idiopathic and unexplained infertility cases, new, easy-to-use OS indicators are important to provide a diagnosis. Becatti and colleagues [12] found that oxidative markers from blood, representing an easy-to-obtain sample, reflect seminal oxidative status. The ROS production of blood leukocytes correlates with semen ROS production and parameters and can be suggested as a new potential and less invasive indicator for male preconception care, in particular for idiopathic infertile patients before in vitro fertilization treatments.

Another debated topic in the field is the effect of leukocytes, found in the semen, on spermatozoa. A concentration of peroxidase positive leukocytes greater than 1 million/mL is defined as leukocytospermia [1]. Leukocytes in semen play key roles in immunosurveillance and in removing abnormal sperm cells, but it is well known that they can be involved in ROS production; in particular, active leukocytes generate up to 100-fold more ROS than inactive leukocytes. Gill et al. [13] proposed a study in this Special Issue designed to verify the relationship between male infertility, semen parameters, sperm DNA fragmentation, and oxidation reduction potential in semen and leukocytospermia. The authors confirmed a strong relationship among male infertility, sperm DNA fragmentation and OS; however, no relations with these variables and leukocytospermia were found, indicating that leukocytospermia is not necessarily the only cause of OS in human semen.

## 3. Studies on Animal Models

The contribution that animal models have had on human research is undeniable, and this holds true in the field of male infertility. In the present Special Issue, many aspects have been considered when using animal models, starting from compounds that cause OS, antioxidants for OS treatment, and the administration of different diets rich in pro-oxidant and antioxidant substances.

The research of Hug and colleagues [14] deals with the crucial role of environmental chemicals, particularly endocrine-disrupting chemicals (EDCs), as contributors to the progressive decline in semen quality and sperm DNA integrity [15]. The focus of the study was on mice’s epididymal compartment, where spermatozoa are stored and acquire their functional role. The authors found that EDCs did not have any effect on sperm motility, vitality, and the acrosomal integrity of mice epididymal spermatozoa. However, they had a detrimental impact on the integrity of the sperm nucleus and DNA structure, probably due to the pro-oxidant properties of the EDCs, which generate an excess of ROS, mitigated by co-administration of antioxidant formulation. The authors hope that these results, if confirmed and translated to humans, can become part of the standard care before ART treatments.

Two other studies published in the Special Issue were performed on rabbit bucks [16,17]. The rabbit buck is a valuable model to study mature spermatozoa because the semen can be easily and continuously collected using an artificial vagina without the sacrifice of the animal.

Mattioli and colleagues [16] administered two different diets to rabbit bucks, one rich in a pro-oxidant agent (flaxseed oil) and the other in a pro-atherogenic compound (coconut oil) and evaluated their effects on blood and semen. While sperm kinetics and viability were decreased only in the group fed with a high-atherogenic diet, the trend in the oxidation markers and cytokines in both groups underlined the close relation between inflammation and oxidation. The authors suggested the importance of antioxidant protection administered concomitantly to a polyunsaturated fatty acid (PUFA)-enriched diet in order to minimize the negative effects of oxidation and promote the benefits of n-3 PUFAs.

The role of n-3 PUFAs is a fascinating topic in male fertility, and the relevance of the n-3/n-6 PUFA ratio is well known. However, further investigations on n-3 PUFA dietary supplements, as proposed by Mattioli, are needed. In fact, it is necessary to consider the susceptibility of this type of molecule to oxidation and the possibility that n-3 PUFA supplementation becomes a source of oxidized products. Therefore, the nature, quality, and quantity of any supplements with n-3 PUFAs must be carefully evaluated, as well as the possibility of integrating them with additional antioxidants, in order to obtain the optimal balance of the well-recognized antioxidant and anti-inflammatory properties of this category of PUFAs with respect to their intrinsic susceptibility to oxidation.

The rabbit was also proposed as a model to investigate the effect of the goji berry (GB, rich in antioxidant compounds), used as a supplementation of the standard diet, on the semen traits, antioxidant capacity of seminal plasma, and histological features of the reproductive tract [17]. The dietary administration of the GB improved several semen traits and the histological and functional characteristics of the testis and epididymis. On the contrary, antioxidant and anti-inflammatory parameters were unaffected by the diet. Natural and nutraceutical products are a class of safe to use compounds and free of specific negative side effects. The GB in particular can be proposed as a new ingredient for rabbit feed as an innovative strategy to increase the welfare and fertility of the animals.

Finally, Arafat and colleagues [18] proposed the locust (*Locusta migratoria*), a migratory insect, as a model to investigate the nanotoxicological influence of aluminum oxide nanoparticles. This is an extremely important indicator, since a significant amount of research is available on the importance and broad applications of aluminum nanoparticles [19,20].

For this reason, studying the bioaccumulation of these compounds in living organisms is a relevant issue. In addition, in the same study, the authors used a whole-body extract of American cockroaches (*Periplaneta Americana*, PAE) to attenuate nanoparticle-induced toxicity. The literature reports that PAE exerts therapeutic effects in inflammatory disorders and it is used in clinical practice. PAE is a mixture containing polyols, dopamine, peptides (such as thymosin, dipeptide, and antimicrobial peptides), nucleosides, viscous sugar amino acids, amino acids, coumarin, epidermal growth factors, and other unclear active components [21]. The authors found that aluminum nanoparticles penetrate into the blood–testis barrier and accumulate in the testis. This phenomenon causes overproduction of ROS, triggering DNA damage and cell apoptosis. Pretreatment with PAE counteracts the detrimental effects of nanoparticles in the testes. The study is original and suggests potential applications of PAE in preventing testicular impairment and the conceivable utilization of locusts for nanotoxicology studies.

## 4. In Vitro Applications

In vitro applications are a relevant research area related to OS and antioxidants. It enables one to acquire scientific data in a controlled environment, avoiding confounding variables and without the involvement of humans and animals.

The review of Moretti and colleagues [5] is an in vitro study using spermatozoa as a cell model to assess the impact of antioxidants against OS; spermatozoa are an ideal model because of their intrinsic characteristics and the ease of collection. The in vitro standardization of this kind of research requires the evaluation of several endpoints such as sperm motility and vitality, the status of the acrosome and DNA, and the membrane mitochondrial potential.

The literature on the use of human spermatozoa as an in vitro cellular model to test antioxidant compounds is extensive and it arises from the necessity to protect spermatozoa from OS during semen handling and the use of ARTs. The capacity of antioxidant compounds to defend against OS induced in human sperm has been studied extensively in vitro, and numerous other studies have successfully supplemented media used in human and animal semen cryopreservation with antioxidants [5,22,23,24].

Studying the best antioxidants and their concentrations for utilization as in vitro supplement of media used for semen handling is an open field of research. The majority of the research in this area is mainly observational, and new insights into the mechanism of action of the various compounds are necessary. The review included in this Special Issue represents a summation of the effects of different antioxidants on human spermatozoa, including polyphenols and phytochemical and natural extracts, often obtained from waste materials, thus stimulating a circular, sustainable economy [25].

The last article from Moreira and colleagues [26] proposes a model of the rodent Leydig cell line to test the toxicity of chromium picolinate and the same insulin-resistant model to test the possible antioxidant/antidiabetic activities of chromium picolinate.

Heavy metals such as chromium are considered EDCs, with known toxicological risks to reproductive health and male fertility by affecting semen quality parameters and the secretory function of accessory sexual glands [27]. Despite this, chromium picolinate has become a very popular supplement to reduce weight or manage blood glucose levels [28] and for many other uses. Chromium picolinate is also known to exert antioxidant effects in cellular systems, such as enhancing the activity of antioxidant enzymes and decreasing damage caused by OS [29,30], but its impact on male fertility raises concerns.

The results of the study published in this Special Issue cautioned the use of chromium picolinate at high concentrations, and its antidiabetic properties were not observed in the insulin-resistant model of Leydig cells, even though an alleviation of OS in Leydig cells was observed. The significance of this research is relevant because it focuses on the safety of chromium picolinate for the reproductive health of men.

Ending this Editorial, we would like to acknowledge the authors that have contributed to this Special Issue “Antioxidants in Male Human and Animal Reproduction: In Vitro and In Vivo Studies”. They provided outstanding contributions and highlighted the relevance of OS in the field of male infertility and the possible use of antioxidants for many purposes, such as when directly administered to patients and animals or used in vitro during semen manipulation.
